# An Algorithm for Rapid and Low-Cost Detection of Carbapenemases Directly from Positive Blood Cultures Using an Immunochromatographic Test

**DOI:** 10.3390/antibiotics15010001

**Published:** 2025-12-19

**Authors:** Patricia del Carmen García, Pamela Rojas, Ana María Guzmán, Sofía Paz Torres, Aniela Wozniak

**Affiliations:** 1 Department of Clinical Laboratories, School of Medicine, Pontificia Universidad Católica de Chile, Santiago 8331150, Chile; pgarciacan@uc.cl (P.d.C.G.); aguzmand@uc.cl (A.M.G.); sofa.torres@uc.cl (S.P.T.); 2 Laboratory of Microbiology, Clinical Laboratories Service, UC-CHRISTUS Health Network, Santiago 7820436, Chile; 3 Multidisciplinary Initiative for Collaborative Research on Bacterial Resistance, MICROB-R, Santiago 7550000, Chile; projas@hurtadohosp.cl; 4 Padre Hurtado Hospital, Santiago 1340000, Chile; 5 Laboratory of Biochemistry, Clinical Laboratories Service, UC-CHRISTUS Health Network, Santiago 7820436, Chile

**Keywords:** immunochromatographic test, carbapenemase detection, blood culture, NDM, OXA-48, confirmatory testing

## Abstract

**Background/Objectives**: Detection of carbapenemases (KPC, OXA-48, VIM, IMP, NDM) from blood cultures (BCs) by standard methods takes 48–72 h and includes BC seeding, susceptibility testing and carbapenemase detection. Automated qPCR panels provide results in 1 h but are very costly. We aim to evaluate a low-cost and rapid immunochromatographic (IC) test directly from positive BCs using the reference method as a comparator. **Methods**: Ninety-one positive BCs from real-world patients and sixty-four simulated BCs were included. BC broth was treated with SDS and washed before analysis with the K.N.I.V.O. carbapenemase detection IC test. Discordant results were confirmed through the NG Carba-5 IC test and GeneXpert Carba-R qPCR test. **Results**: The test detected 100% of the 87 carbapenemase-producing BCs tested (sensitivity: 100% [CI95%: 95.8–100%]). However, 13 BCs generated false positive bands for NDM and/or OXA-48 (specificity: 80.8% [CI95%: 69.5–89.4%). The positive and negative predictive values were 87.0% (CI95%: 80.4–91.6%) and 100% (CI95%: 93.5–100%). Analysis of BCs providing false positive results through both confirmatory tests showed that BCs were negative for these carbapenemases. **Conclusions**: This is the first evaluation of the K.N.I.V.O. IC test directly from positive BCs, with a pragmatic confirmation algorithm using a second IC test or qPCR in case of NDM or OXA-48, that addresses K.N.I.V.O.’s specificity gap. The main limitation of this work is that confirmatory testing was performed only in false positives. The implementation of the K.N.I.V.O. IC test would contribute to early carbapenemase detection in BCs and is an alternative for low-resource hospitals where qPCR panels are not available.

## 1. Introduction

Bloodstream infections (BSIs) and associated serious conditions like sepsis and septic shock are among the top causes of mortality worldwide, killing between one in three and one in six of affected patients [1]. BSIs are associated with extended hospital stay, increased use of antimicrobials, and a major economic burden [2]. One of the most important factors determining the outcome of BSIs is the time to appropriate antimicrobial treatment. Observational data reported a 30% increase in mortality when adequate treatment was not administered within the first 24 h [3]. For this reason, broad-spectrum antimicrobials are usually administered as empirical treatment in patients with BSIs while waiting for final microbiological reports [4]. This may lead to the overuse of broad-spectrum antimicrobials and its associated side-effects, including enhancement of antimicrobial resistance [5], and increased costs, especially in developing countries.

BSIs caused by carbapenemase-producing bacteria pose a significant challenge for the management of BSIs because they have a higher mortality rate than those caused by carbapenem-susceptible bacteria [6]. KPC, OXA-48, VIM, NDM and IMP are the most frequent carbapenemases, referred to as the “big five”, and confer resistance to a broad range of antimicrobials including penicillins, cephalosporins and carbapenems, the latter being often used a last resort for severe infections. A study involving carbapenem-resistant bacteria obtained from blood cultures (BCs) between January 2018 and April 2024 among 11 hospitals in Chile showed that 2626 were *Enterobacterales* (83%) and 533 were *P. aeruginosa* (17%). Fourteen percent of *Enterobacterales* produced NDM, KPC and VIM, and 43% of *P. aeruginosa* were producers of VIM and KPC (unpublished data from the MICROB-R Network in Chile).

Detection of carbapenemase-producing bacteria from BCs by the reference method usually takes 48–72 h [4]. The BCs are subcultured in appropriate media, and after 18–24 of growth, identification is performed through MALDI-TOF (Bruker, Bremen, Germany) mass spectrometry. Susceptibility testing takes another 18–24 h and carbapenem-non-susceptible bacteria are analyzed through Carba-NP test for biochemical detection of carbapenemase activity. Finally, the enzyme is identified using an immunochromatographic (IC) test to detect the “big five” carbapenemases. The FilmArray blood culture ID panel (BCID2) (BioFire, Salt Lake City, UT, USA) detects 43 different targets, including the “big five” carbapenemases, directly from positive BCs in 1 h. However, its main hurdle is the elevated cost [7]. Similarly, the GeneXpert Carba-R (Cepheid, Sunnyvale, CA, United States) platform is an automated q-PCR that detects the “big-five” carbapenemase genes in rectal swabs and has shown high sensitivity and specificity (100%) when used directly from positive BCs [8]. Unfortunately, the GeneXpert test is also very expensive. Although IC tests for carbapenemase detection are standardized to be performed with pure bacterial suspensions, they were evaluated directly in positive BCs: the NG Carba-5 IC test (NG-Biotech, Marcy l’Étoile, France) [9,10,11,12] and Resist-5 O.K.N.I.V. (CORIS Bio-concept, Gemblouxm Belgium) IC test [13,14] are the most reported ones. In the present work, we evaluated the K.N.I.V.O. (which stands for KPC, NDM, IMP, VIM, OXA-48) carbapenemase detection IC test (Genobio, Tianjin, China) directly in positive BCs from both simulated and real-world patients, using an in-house-developed method for BC preparation prior to IC testing, using the reference method as a comparator.

## 2. Results

### 2.1. Performance of K.N.I.V.O. IC Test

Ninety-one positive BCs from real-world patients and sixty-four simulated BCs were included in this study (total = 155 BCs). Distribution of species and carbapenemases produced by the different isolates is shown in Table 1.

**Figure 1:**
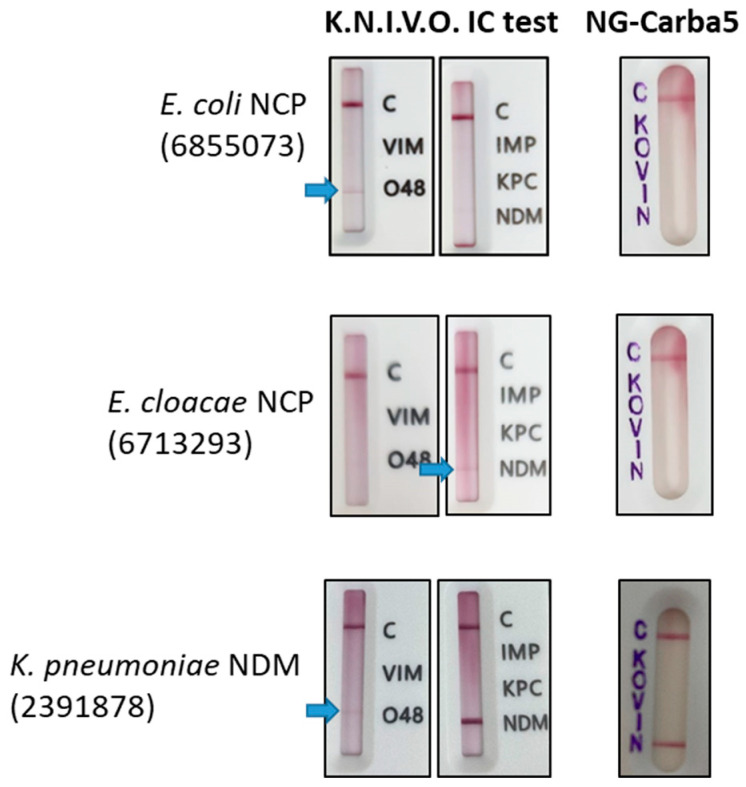
Analysis of discordant results. Representative results of 3 BCs exhibiting false positive bands (blue arrows) are shown. NCP: non-carbapenemase-producing. C: control band; K: KPC; O: OXA-48; V: VIM; I: IMP; N: NDM.

On the other hand, 87 BCs had carbapenemase-producing bacteria and 68 had non-carbapenemase-producing (NCP) bacteria. The K.N.I.V.O. IC test detected 100% of the 87 carbapenemase-positive BCs, including 30 NDM, 16 KPC, 24 VIM and 4 OXA-48 and 13 double-carbapenemase producers (Table 1). No BCs with IMP carbapenemase were analyzed because of the low prevalence of this carbapenemase in Chile. Therefore, this carbapenemase was not included in our study. According to these results, the direct IC test for detection of carbapenemases in positive BCs had a global sensitivity of 100%. However, 13 BCs generated false positive bands in the screening IC assay: 3 for NDM, 5 for OXA-48 and 5 for NDM + OXA-48, meaning that the global specificity of the test was 80.8% (Table 2). The positive and negative predictive values were 87.0% and 100%, respectively. Non-specific bands were weak (Figure 1); in contrast, bands generated by true OXA-48 and NDM isolates had the same intensity as the control band. False positives occurred in *K. pneumoniae* (*n* = 4), *E. coli* (*n* = 5), *E. cloacae* (*n* = 1), *S. marcescens* (*n* = 1) and *M. morganii* (*n* = 1). Of the 13 false positives, 12 occurred in real-world BCs whereas only 1 occurred in a simulated BC. The sensitivity, specificity, positive and negative predictive value were calculated for each carbapenemase as shown in the lower part of Table 2. Sensitivity was 100% for the four carbapenemases analyzed. Specificity values for VIM and KPC were 100%. In contrast, specificity values for NDM and OXA-48 were 92.8% and 93.3%, respectively.

BCs showing discordant results between the reference method and K.N.I.V.O. IC test directly from BC were analyzed using the NG Carba-5 IC test and GeneXpert Carba-R. Confirmatory testing of false positive samples with NG Carba-5 IC and GeneXpert Carba-R was negative for OXA-48 and NDM, indicating that the weak bands observed were non-specific (Table 3, Figure 1).

### 2.2. Bacterial Load of Blood Cultures

**Figure 2:**
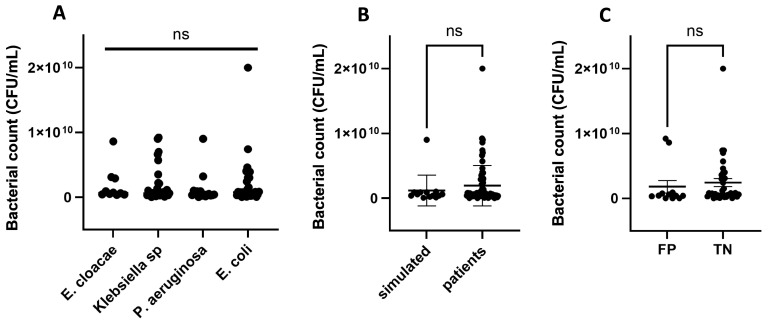
Bacterial counts of BCs that were positive for different species (**A**), bacterial counts of BCs obtained from patients compared to simulated ones (**B**), bacterial counts of BCs that exhibited false positive (FP) results compared to the true negative (TN) ones (**C**). Mean of data groups were compared using the ANOVA test and Student’s *t* test. ns: not significant.

Because non-specific bands were observed in the optimization step when using 1800 µL of BC fluid (see Materials and Methods), we hypothesized that false positive results could arise from a high bacterial count. Bacterial counts of BCs were between 10^8^ and 10^9^ CFU/mL and they were not different among the different species nor among simulated and real-world BCs (Figure 2A,B). Furthermore, samples exhibiting false positive results did not have higher bacterial counts than true negative samples (Figure 2C).

**Figure 3:**
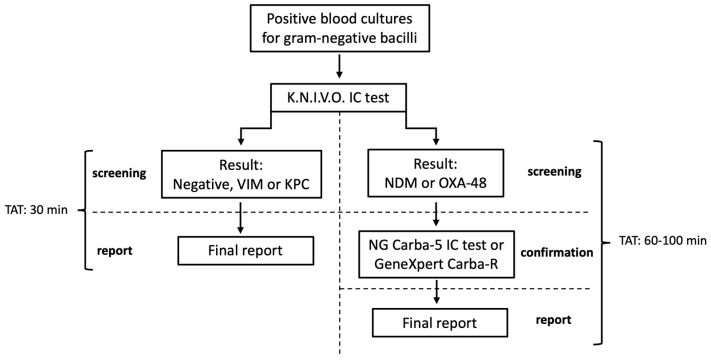
Algorithm for rapid and low-cost detection of the “big five” carbapenemases directly from BCs. Only BCs positive for Gram-negative bacilli upon microscopical observation of Gram-stained smears are analyzed using this algorithm. IC test results that are negative or positive for VIM or KPC can be given immediately, whereas positive results for NDM or OXA-48 should be confirmed through the NG Carba5 IC test or GeneXpert CARBA-R qPCR. TAT: Turn-around time.

In view of the results obtained, we propose an algorithm for early detection of carbapenemases in positive BCs for Gram-negative bacilli, using the K.N.I.V.O. IC test followed by a confirmatory test in case the results are NDM and/or OXA-48 (Figure 3). The total turn-around time (TAT) for negative, KPC and VIM results is 30 min. In the case of NDM and OXA-48, which require confirmatory testing, the total turn-around time is 60–100 min. Confirmation of NDM and OXA-48 may be performed through the NG Carba-5 IC test in low-resource settings (30 min for screening IC test + 30 min for confirmatory IC test), or through GeneXpert Carba-R in higher-resource settings (30 min for screening IC test + 70 min for confirmatory GeneXpert Carba-R).

## 3. Discussion

Early adequate antimicrobial therapy in cases of sepsis or septic shock is crucial to improve patient outcome. Empirical treatment should be broad enough to cover possible pathogens according to local epidemiology data [4]. Ceftazidime/avibactam (CZA) is a last-generation β-lactam/β-lactamase-inhibitor combination, widely used as an empirical therapy in BSIs in regions with a high prevalence of carbapenemase-producing organisms [15], like Chile. Class A and D enzymes like KPC and OXA-48 are inhibited by avibactam, whereas class B enzymes like NDM or VIM are not, and so other options like aztreonam/CZA may be used [16]. Because bacterial species and carbapenemase class are not known at the time of empirical treatment prescription, the antibiotics prescribed may not be adequate [17]. For this reason, once laboratory results are available, they must be used to adjust the initial empirical treatment. The procedure evaluated here can determine the carbapenemase class 48–72 h earlier than the reference method. Knowing carbapenemase class before standard tests are ready would avoid unnecessary overuse of broad-spectrum antimicrobials like CZA in case no carbapenemases are detected, and consequently would prevent or delay the emergence of antimicrobial resistance towards last-resort antimicrobials like CZA. Importantly, in case a class B enzyme is detected, the early diagnostic would support an empiric therapy with aztreonam/CZA instead of CZA alone; in this case, early diagnosis would also contribute to patient survival.

Most reports of IC tests for early diagnosis of carbapenemases in BCs mainly include *Enterobacterales* [10,12,13,14], with few reports in *P. aeruginosa* and using a very low number of isolates (two IMP producers and eleven NCP *P. aeruginosa* isolates) [9,11]. Our results show that this test can detect carbapenemase-producing *P. aeruginosa*, with 100% (95% CI: 95.8–100%) sensitivity using the reference method as a comparator. Although our method achieved an excellent sensitivity for detection of the “big five” carbapenemases, NDM and OXA-48 showed low specificity (80.8%; CI 95%: 69.5–89.4%). Most reports about the evaluation of IC tests to detect carbapenemases directly from BCs used the NG Carba-5 IC test and reported overall sensitivities that ranged from 93% to 100% [9,10,11,12]. In these reports, the IC test failed to detect some VIM and/or NDM carbapenemases, whereas all KPC and OXA-48 were detected. A recent work compared NG Carba-5 with the K.N.I.V.O. IC test using different inoculum sizes of bacterial suspensions as recommended by the manufacturer and reported false positive results for OXA-48 and NDM using the K.N.I.V.O. IC test with all inoculum sizes [18]. In contrast, the NG Carba-5 test showed false positive results only when a heavy inoculum was used. These results suggest that whereas the K.N.I.V.O. IC test seems to have improved sensitivity for the detection of VIM and NDM, it has lower specificity for detection of NDM and OXA-48. To our knowledge, this is the first evaluation of the K.N.I.V.O. IC test applied directly to positive BCs, with a pragmatic confirmation algorithm that addresses K.N.I.V.O.’s specificity gap. We observed that false positive results occurred in BCs having similar bacterial counts to the true positive samples, suggesting that inoculum size is not the problem. False positives occurred in almost all species tested (Supplementary Material, Table S1), indicating that species is not the problem either, and suggesting that particular bacterial traits may be responsible for these non-specificities. False positives in lateral flow assays may arise due to sub-optimal composition of lysis buffers, non-specific protein–conjugate interactions and cross-reactivity [19].

The limitations of this work include the fact that only two health networks were included. In addition to this, because the NG Carba-5 IC test and GeneXpert Carba-R were performed only in samples exhibiting false positive results, a partial-verification bias may have occurred. In addition, only five BCs with an OXA-48-producing organism were tested, basically due to the low prevalence of this carbapenemase in our setting, and IMP carbapenemase was not tested. The multi-site workflow may be another limitation of this work, because real patients’ BCs that became positive during the weekend were stored before testing whereas simulated BCs were not. However, false positive bands were not generated by BCs that had been stored over the weekend; they were generated by BCs that were analyzed immediately or within 2–3 h. Validation of the algorithm proposed in real-world conditions would be required to use this test in routine analysis.

Screening positive BCs with the K.N.I.V.O. IC test plus mandatory confirmation for NDM/OXA-48 positives delivers rapid and implementable stewardship value in routine BC workflows. Because of its low cost and short turn-around time, it may be an alternative for rapid and low-cost detection of carbapenemases directly from BCs in low-resource hospitals, where automated qPCR panels are not available.

## 4. Materials and Methods

### 4.1. Blood Cultures from Patients

The real-world positive BCs were obtained from patients hospitalized during 2024 and 2025 in the Hospital of UC-CHRISTUS Health Network and in the Padre Hurtado Hospital, both in Santiago, Chile. BCs were analyzed using the reference method as follows. After being incubated in a BACT/ALERT VIRTUO™ system (Biomerieux, Marcy l’Étoile, France) and flagged positive, BCs were Gram-stained, microscopically observed and cultured in appropriate media. Colonies obtained were identified using MALDI-TOF spectrometry analysis in UC-CHRISTUS Hospital, and Phoenix^TM^ (Beckton-Dickinson, Sparks, MD, USA) in Padre Hurtado Hospital. Antimicrobial susceptibility testing was performed through the agar dilution method and those isolates that were non-susceptible (intermediate or resistant) to at least one carbapenem (imipenem, meropenem, ertapenem) were analyzed for carbapenemase production through the Carba-NP test according to CLSI guidelines (Clinical and Laboratory Standards Insitute 2024) [20]. Isolates exhibiting a positive Carba-NP test were analyzed to determine the identity of the carbapenemase/s produced through the K.N.I.V.O. Carbapenemase-Detection IC test (Genobio, Tianjin, China; LOT: 231103) in UC-CHRISTUS Hospital and Resist-5 O.K.N.I.V. (CORIS Bio-concept, Gembloux, Belgium) in Padre Hurtado Hospital. The technician was blinded to the result of the IC test that was performed directly from BCs 2–3 days prior.

### 4.2. Simulated Blood Cultures

Simulated BCs were inoculated with known carbapenemase-producing isolates that were stored in the laboratory, previously confirmed through PCR followed by Sanger sequencing [21] or through the IC test as described above. Isolates were grown in blood agar and used to prepare a McFarland 0.5 bacterial suspension that was diluted 1/100,000 in PBS, and 100–300 µL were added to BACT/ALERT (Biomerieux, Marcy l’Étoile, France) pediatric BC bottles. Using this inoculation protocol, time to positivity was similar to real BCs (9–16 h) [12]. All the bottles were inoculated with 5–10 mL of blood from healthy donors (the authors AW, AMG and ST). Bottles were incubated in a BACT/ALERT VIRTUO™ system until flagged positive and immediately cooled at 4 °C until processing. Time to processing varied between 1 and 10 h depending on the hour that bottles were flagged positive. The technician that read the IC tests was blinded to the species and carbapenemases produced by the isolates inoculated in the BC bottles.

### 4.3. Direct IC Test of BCs

**Figure 4:**
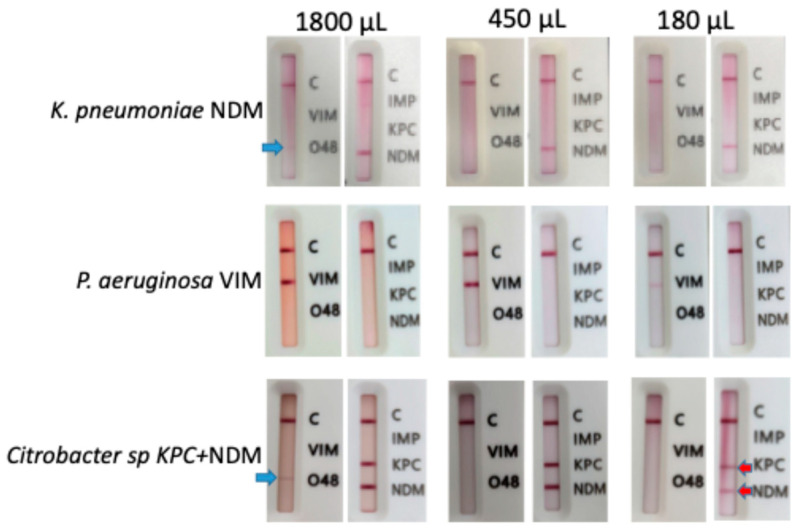
Optimization of the blood culture volume used for direct IC test using three carbapenemase-producing isolates (*K. pneumoniae* NDM, *P. aeruginosa* VIM, *Citrobacter* sp KPC + NDM). Three different BC volumes were used to perform the IC test: 1800 µL, 450 µL and 180 µL. Non-specific bands were observed for OXA-48 when 1800 µL of BC was used (blue arrows). Weak bands were observed for VIM and KPC when 180 µL of BC was used (red arrows). C: Control band of the IC test.

Positive BCs from patients that exhibited Gram-negative bacilli in the microscopic observation were immediately analyzed using the K.N.I.V.O. carbapenemase detection IC test using the protocol as follows. Patient BCs that were flagged positive during the weekend were stored at 4 °C and processed on the next working day. An aliquot of 450 µL of the positive BC was obtained from the bottle and mixed with 50 µL of 10% SDS to lyse blood cells, vortexed and incubated for 5 min at room temperature. Tubes were centrifuged 3 min at 13,000× *g* and supernatant was discarded. The pellet was washed with 1 mL of distilled water and centrifuged again. Supernatant was discarded and the pellet was resuspended in 5 drops of lysis reagent of the K.N.I.V.O. carbapenemase detection IC test, and 50 µL of this mix was added to the sample window of the IC test cassette. Results were read within 15 min. The turn-around time for this test is 30 min (25–45 min). To determine the optimal volume of BC fluid to be used in the direct IC test, 6 simulated BCs of isolates producing KPC, VIM and NDM were evaluated using three different volumes of BC: 180 µL, 450 µL and 1800 µL. Proportional volumes of 10% SDS and water were added to the tubes. The pellets were resuspended in 5 drops of lysis reagent and the procedure was continued as described above. When 180 µL was used, KPC and NDM bands were clearly observed. In contrast, VIM bands had a lower but still readable intensity. When 1800 µL was used, non-specific weak bands were observed for OXA-48 and NDM. When 450 µL was used, bands had similar intensity to the control band, and non-specific bands were not observed (Figure 4). Based on these results, the 450 µL volume was used for subsequent IC tests.

### 4.4. Bacterial Count of BCs

A total of 100 µL of a 1/100,000 dilution of positive BCs was cultured in McConkey agar to quantitatively determine the bacterial load in simulated and real-world BCs. After 24 h of incubation at 35 °C, colonies were counted.

### 4.5. Analysis of Discordant Results

Discordant results (inconsistent with reference method, i.e., false positives) were analyzed with the NG Carba-5 IC test (NG-Biotech, Guipry, France; LOT: 230515-01-B) using the same sample preparation protocol described above. The turn-around time for this test is also 30 min (25–35 min). Discordant results were also analyzed through GeneXpert Carba-R (Cepheid; LOT: 1001425237) using the protocol described by Cointe and coworkers [8]. Briefly, 40 µL of positive blood culture fluid was mixed with 1700 µL of GeneXpert sample reagent buffer and incubated for 10 min at room temperature. Using a disposable plastic pipette, 1.5 mL was loaded into the cartridge and analyzed in the GeneXpert equipment. The turn-around time for this test is 75 min (70–80 min). To ensure reproducibility of the results, the tests were performed by technicians with >3 years of experience in laboratory microbial diagnosis.

### 4.6. Statistical Analysis

All statistical analyses were carried out using GraphPad Prism 10 v 10.6.1. One-way ANOVA analyses were performed when 3 or more variables were evaluated. Tukey’s test was used for multiple comparisons. Student’s *t*-test was used to compare two variables. For all analyses, a *p* value = 0.05 was considered statistically significant.

## Figures and Tables

**Table 1:** Isolates included in this study.

	Nº of Isolates (Simulated + Patients)								
	NDM	KPC	VIM	OXA48	NDM + VIM	KPC + NDM	OXA48 + NDM	NCP	Total
*Escherichia coli*	1 + 0	2 + 0	0 + 0	2 + 0	0 + 0	0 + 0	0 + 0	1 + 36 ^&^	6 + 34
*Klebsiella pneumoniae*	5 + 8	3 + 2	0 + 0	2 + 0	0 + 0	0 + 4	1 + 0	0 + 11	11 + 25
Other *Klebsiella* species ^$^	1 + 0	1 + 0	0 + 0	0 + 0	0 + 0	3 + 2	0 + 0	0 + 3	5 + 5
*Enterobacter cloacae*	2 + 4	3 + 0	2 + 0	0 + 0	1 + 0	0 + 0	0 + 0	0 + 3	8 + 7
Other *Enterobacterales* *	7 + 0	0 + 0	0 + 0	0 + 0	0 + 0	2 + 0	0 + 0	0 + 4	9 + 4
*Pseudomonas aeruginosa*	0 + 0	4 + 1	17 + 5	0 + 0	0 + 0	0 + 0	0 + 0	2 + 5	22 + 11
Other non-fermenters ^#^	2 + 0	0 + 0	0 + 0	0 + 0	0 + 0	0 + 0	0 + 0	0 + 3	2 + 3
Total	18 + 12	13 + 3	19 + 5	4 + 0	1 + 0	5 + 6	1 + 0	3 + 65	64 + 91

NCP: Non-carbapenemase producer; ^&^ two BCs also showed K. pneumoniae; ^$^ *K. variicola*, *K. aerogenes*, *K. oxytoca*; * *Citrobacter* sp., *Raoultella ornithinolytica*, *Serratia marcescens*, *Morganella morganii*; ^#^ *Pseudomonas putida*, *Stenotrophomonas maltophilia*, *Moraxella osloensis*, *Proteus hauseri*, *Acinetobacter baumannii*.

**Table 2:** Performance of detection of carbapenemases in positive BCs.

					Global Values * % (95% CI)			
	TP	FP	FN	TN	Sens	Spec	PPV	NPV
BCs	87	13	0	55	100% (95.8–100)	80.8% (69.5–89.4)	87.0% (80.4–91.6)	100% (93.5–100)
					**Values for Each Carbapenemase** **% (95% CI)**			
	**TP**	**FP**	**FN**	**TN**	**Sens**	**Spec**	**PPV**	**NPV**
NDM	43	8	0	104	100% (91.8–100)	92.8% (86.4–96.8)	84.3% (73.4–91.3)	100% (96.5–100)
VIM	25	0	0	130	100% (86.3–100)	100% (97.2–100)	100% (86.3–100)	100% (97.2–100)
KPC	27	0	0	128	100% (87.2–100)	100% (97.2–100)	100% (87.2–100)	100% (97.2–100)
OXA48	5	10	0	140	100% (47.8–100)	93.3% (88.1–96.8)	33.3% (21.5–47.6)	100% (97.4–100)

TP: true positive; FP: false positive; FN: false negative; TN: true negative; Sens: sensitivity; Spec: specificity; PPV: positive predictive value; NPV: negative predictive value. * IMP was not included in the evaluated set.

**Table 3:** BCs exhibiting false positive results (*n* = 13).

Isolate ID	Species	Reference Method	K.N.I.V.O. Direct from BC	NG-Carba5 Direct from BC	GeneXpert Carba-5
2391878	*K. pneumoniae*	NDM	NDM + OXA-48	NDM	NDM (CT = 14.3)
6992617	*K. pneumoniae*	negative	NDM	negative	negative
7001399	*K. pneumoniae*	negative	OXA-48	negative	negative
7018929	*K. pneumoniae*	negative	NDM + OXA-48	negative	negative
2392714	*P. aeruginosa*	VIM	VIM + OXA-48	VIM	VIM (CT = 25.1)
6888910	*E. coli*	negative	NDM + OXA-48	negative	negative
6899532	*E. coli*	negative	OXA-48	negative	negative
6855073	*E. coli*	negative	NDM + OXA-48	negative	negative
6897157	*E. coli*	negative	NDM + OXA-48	negative	negative
8141881	*E. coli*	negative	NDM	negative	negative
7015779	*S. marcescens*	negative	NDM + OXA-48	negative	negative
6915699	*M. morganii*	negative	OXA-48	negative	negative
6713293	*E. cloacae*	negative	NDM	negative	negative

## Data Availability

Data supporting the reported results can be found in Supplementary Data Table S1.

## Supplementary Materials

The following supporting information can be downloaded at: https://www.mdpi.com/article/10.3390/antibiotics15010001/s1, Table S1: Complete data for simulated and patient´s BCs.

## Abbreviations

The following abbreviations are used in this manuscript:

BSI	bloodstream infection
BC	blood culture
NCP	non-carbapenemase producer
ns	not significant
TP	true positive
TN	true negative
FP	false positive
FN	false negative
PPV	positive predictive value
NPV	negative predictive value
TAT	turn-around time
